# HBV reactivation under immunosuppressive treatment – a case series

**DOI:** 10.1186/1471-2334-14-S7-O11

**Published:** 2014-10-15

**Authors:** Mihaela Rădulescu, Anca-Ruxandra Negru, Cristina Popescu, Violeta Molagic, Daniela Munteanu, Raluca Mihăilescu, Cătălin Tilişcan, Alina Lobodan, Irina Lăpădat, Smaranda Gliga, Georgiana Jugănaru, Aida Adamescu, Victoria Aramă

**Affiliations:** 1 National Institute for Infectious Diseases "Prof. Dr. Matei Balş", Bucharest, Romania; 2 Carol Davila University of Medicine and Pharmacy, Bucharest, Romania

## Background

Inactive HBV carriers, under immunosuppressive treatment for malignancies or rheumatologic diseases, have an increased risk for HBV reactivation. HBV reactivation under immunosuppression has a high rate of acute liver failure and death. HBV screening is mandatory for patients with hematological malignancies or rheumatologic diseases who are due to receive immunosuppressive treatment.

## Methods

We retrospectively analyzed 13 patients from Department 3 of the National Institute for Infectious Diseases "Prof. Dr. Matei Balş" that were diagnosed with HBV reactivation under immunosuppressive treatment for different malignancies or rheumatologic diseases.

## Results

Thirteen patients were enrolled, 6 women and 7 men, with a median age of 56 years [32-70.5]. Seven patients were diagnosed with hematologic malignancies (6 with non-Hodgkin lymphoma and 1 with chronic lymphatic leukemia), 4 with rheumatologic diseases (1 with ankylosing spondylitis, 1 with Reiter syndrome and 2 with rheumatoid polyarthritis), 1 patient with ovarian cancer and 1 patient with renal transplant. Of 13 patients, one had negative HBsAg and protective HBs antibodies titers, before immunosuppressive treatment, with further HBs retroseroconversion.

On admission, TGP median value was 1,125 IU/dL [491-1738]. Median prothrombin concentration on admission was 85% [68-101] and median nadir of prothrombin concentration was 66% [54-84].

Median hospitalization period was 20.77 days. Hospitalization period was directly correlated with ALT and AST values at the time of admission (p=0.06 respectively p=0.015) and negatively correlated with prothrombin concentration at admission (p=0.059) and lymphocytes number (p=0.058).

Ten patients were treated with entecavir and three with lamivudine. Three patients died, all of them with hematologic malignancies – two deaths were due to hematologic disease and one due to liver failure. Median age of deceased patients was 73 years [68.0-75.0], while in the surviving group the median age was 50 years [27.7-58.5] (p=0.049).

## Conclusion

In HBV reactivation patients, older age is a risk factor for mortality. Higher liver transaminases and lower prothrombin concentration and lymphocytes are associated with longer hospitalization period. Even patients with negative HBs antigen can reactivate HBV infection during immunosuppression, requiring close monitoring.

